# Determinants of recovery time from cholera in a conflict-affected agro-pastoral region of ethiopia: evidence from survival models

**DOI:** 10.1186/s13031-026-00818-w

**Published:** 2026-06-23

**Authors:** Tegenu Tento, Sebisibe Kumaso, Sitotaw Ahmed, Tilahun Asena, Nurhussen Ahmed

**Affiliations:** 1 Department of Statistics, College of Natural and Computational Sciences, Jinka University, Jinka, Ethiopia; 2 Health Monitoring and Evaluation Department, Ale Zone, Kolango, Ethiopia; 3 Division of Epidemiology and Biostatistics, Wits School of Public Health, University of Witwatersrand, Johannesburg, South Africa; 4 Global Change Institute, University of Witwatersrand, Johannesburg, South Africa; 5 Department of Surgery, Yekatite 12 Hospital Medical College, Addis Abeba, Ethiopia

**Keywords:** Agro pastoral, Conflict, Exposure recovery time, Survival analysis

## Abstract

**Background:**

Cholera remains a major public health concern in Ethiopia, particularly in conflict-affected agro-pastoral regions where access to healthcare, clean water and vaccination is limited. The main objective was to identify predictors of time to recovery from cholera among patients in a conflict-affected agro-pastoral area of Ethiopia using survival analysis models. Understanding the clinical and epidemiological factors influencing recovery time is critical for improving outcomes and guiding outbreak responses.

**Methods:**

A retrospective cohort study was conducted using data from cholera treatment centers in a conflict-affected agro-pastoral region. Time to recovery was analyzed using Kaplan-Meier recovery curves, Cox proportional hazards, and Accelerated Failure Time (AFT) models. Variables included clinical signs, demographic factors, vaccination status, and exposure history.

**Results:**

Severe dehydration (HR = 0.49, *p* < 0.0001; TR = 1.49), low pulse rate (HR = 0.69, *p* = 0.005), and lack of vaccination (HR = 0.56, *p* < 0.0001) were associated with significantly longer recovery times. Notably, patients with known contact with cholera cases showed slower improvement (HR = 0.63, *p* = 0.0003), suggesting higher exposure or delayed care-seeking. Vaccinated individuals and those with mild dehydration recovered significantly faster. The log-Normal AFT model confirmed these findings, showing prolonged recovery among high-risk groups.

**Conclusion:**

In conflict-affected agro-pastoral settings, clinical severity, vaccination status, and exposure history are key determinants of cholera recovery time. These findings underscore the importance of early case detection, aggressive rehydration, and targeted vaccination, especially among known contacts and high-risk individuals. Public health interventions should prioritize rapid response strategies and contact-based follow-up to reduce disease burden and improve recovery outcomes.

## Background

Cholera is an acute intestinal infection caused by Vibrio cholerae, which can cause severe dehydration and death if not treated in time [1]. Poverty, violence, and natural disasters are all factors that can cause its spread [2]. In Ethiopia, cholera is a major public health concern, and its outbreak has been reported in various areas, while its mortality rate is still higher than the global target [3, 4]. The major risk factors for cholera include a lack of clean water, poor sanitation, and poor hygiene practices [2]. To control cholera, Water, Sanitation, and Hygiene (WASH) improvement, early detection, and timely treatment are essential, but detection and outbreak duration have decreased, while notification times have increased [5, 6].

Every year, cholera causes 1.3-4.0 million new cases and 21,000-143,000 fatalities worldwide [7]. Notably, Africa had the highest Case Fatality Ratio of 1.9% among all areas between 2014 and 2023 [8]. Conflict and displacement, as seen in Sudan, Nigeria, Ethiopia, and the Democratic Republic of the Congo, interrupt health services and increase vulnerability [9]. The fact that the total number of cases is not adequately reported is a major reason why cholera continues to spread [10]. Cholera is frequently reported as acute watery diarrhea due to inadequate diagnostic capabilities in remote areas of Ethiopia and cholera outbreak sensitivity [4]. The identification of high-risk rural clusters permits a timely public health response and focused resource allocation to control and eliminate cholera [11]. However, implementation is hampered by insufficient finance, vaccination shortages in hotspot areas with recurring outbreaks, and significant humanitarian requirements [12].

There has been insufficient progress in the achievement of major milestones, such as water, sanitation, and hygiene [13]. Several studies have pointed out that the major risk factor for cholera is the lack of access to WASH services in the WHO African Region [7, 14, 15]. The use of a vaccine approach coupled with an improvement in WASH services is more effective in reducing the risk of cholera compared to the use of these approaches alone [6, 16–18]. The major challenges to the elimination of cholera in the region include security-related communication barriers, administrative barriers to vital resources, and water scarcity challenges [19–21]. Cholera is a serious infectious disease, yet it is often not given the attention it deserves.

Demographic and clinical factors have been found to have an impact as well. Factors that increased the risk of death from cholera included older age, male gender, residency, severe dehydration, peak rainy season, and outpatients [3, 22]. Persons employed in agriculture or pastoral work had a greater rate of infection [23]. Previous vaccination and lack of previous exposure to cholera were found to increase the rate of survival among persons infected by the disease [24, 25]. The factors that influence recovery time are important in these situations as well. This study used the Cox model to investigate the effect of vaccination, exposure, and vital signs on the recovery from cholera in conflict-affected Ethiopian districts.

## Methods

### Study design and data source

This retrospective study observed cholera patients until they recovered. Before data collection began, the study protocol was approved by the institutional review board. Data were extracted from all patients’ cards within the study period. Following data extraction, data entry, data editing, data coding, and data organization were carried out. R software version 4.2.2 was used to perform descriptive and inferential statistics. Retrospective data were obtained from the Ale zone health office cholera weekly report for the period from January to October 2023. The secondary data on the record of cholera patient care follow-up cards were used in this study.

### Study population and sample

According to the Ethiopian Public Health Institute (EPHI), the total number of cholera cases from January to October 2023 was 853 [26]. The study included all reported cases throughout this time period, resulting in a complete cholera case report.

### Variables: exposure and outcome

Exposure variables were sex (female, male), age(< 1, 1–20, 21–40, 41–60, > 60years), residence (urban, rural), dehydration status (severe dehydration, some or moderate dehydration, no or mild dehydration), vaccination (oral cholera vaccine) status (unvaccinated, vaccinated), vomiting (yes, no), water sources (protected, unprotected water sources), exposure history (contact with suspected acute water diarrhea patient) (yes, no), travel history for active area (yes, no), latrine available (yes, no), age adjusted pulse rate (low, normal, high), systolic blood pressure (low, normal, high), and diastolic blood pressure (low, normal, high). The exposure variables were chosen based on the works of literature, and all were included in the models. The outcome variable was the time to recovery of the patient under follow-up in the Ale zone cholera treatment centers.

#### Operational definitions and variables

##### Dehydration

as the excessive loss of body water due to restricting fluid intake, sweating, diarrhoea, vomiting and certain medications. Dehydration is classified according to water weight loss as mild (1–2%), moderate (5%) and severe (10%).

Oral cholera vaccination status: receipt of at least one dose of an oral cholera vaccine before developing cholera. Patients were categorized as vaccinated (received at least one dose of OCV) or unvaccinated (received no OCV).

##### Latrine

is a toilet or an even simpler facility that is used as a toilet within a sanitation system.

### Statistical analysis

The probability distribution of a survival random variable can be described in a variety of ways. To model survival data, non-parametric, semi-parametric, and parametric models are available. The Kaplan Meier estimator is a standard non-parametric survival function estimator that is used to calculate survival probabilities. It considers any point in time as a succession of steps defined by observed survival and censored times, incorporating information from all available observations, both censored and uncensored [27].

$\:{\widehat{\mathrm{S}}}_{\mathrm{K}\mathrm{M}\left(\mathrm{t}\right)\:\:\:\:=\:\:\:\:\prod\:_{{\mathrm{t}}_{\mathrm{i}<\mathrm{t}}}\left(\frac{{\mathrm{n}}_{\mathrm{i}}\:-\:{\mathrm{d}}_{\mathrm{i}}}{{\mathrm{n}}_{\mathrm{i}}}\right)=\prod\:_{{\mathrm{t}}_{\mathrm{i}}<\mathrm{t}}\left(1-\frac{{\mathrm{d}}_{\mathrm{i}}}{{\mathrm{n}}_{\mathrm{i}}}\right)\:\:\:\:\:\:}$Where, $\:{\mathrm{d}}_{\mathrm{i}}$ is the number of cholera cases who recover at time $\:\left({t}_{i}\right)$ ,$\:\:{\mathrm{n}}_{\mathrm{i}}$ is the number of patients who have not yet recover or the total number of individuals at risk before time $\:{(t}_{i})$.

The Cox proportional hazard model, established by Cox, is one of the most prevalent types of regression models used in survival analysis [28]. As a result, the model is known as a semi-parametric model. $\:\mathrm{h}\left(\mathrm{t},\:\mathrm{X},\:{\upgamma\:}\right)=\:{\mathrm{h}}_{\mathrm{o}}\left(\mathrm{t}\:\right)\mathrm{e}\mathrm{x}\mathrm{p}\left({{\upgamma\:}}_{1}{\mathrm{X}}_{1}+{{\upgamma\:}}_{2}{\mathrm{X}}_{2}+\dots\:{+{\upgamma\:}}_{\mathrm{p}}{\mathrm{X}}_{\mathrm{p}}\right)\:$Where, $\:\:ho\:\left(t\right)$ is the baseline hazard function that characterizes how the hazard function changes as a function of survival time, $\:\mathrm{h}(\mathrm{t},\:\mathrm{X},\:{\upgamma\:})$ the hazard function at time * t* with covariates$\:({X}_{1},{X}_{2},{X}_{3}\dots\:{X}_{p})$ and $\:{\upgamma\:}=\:\left({{\upgamma\:}}_{1},{\:{\upgamma\:}}_{2},\:\:{{\upgamma\:}\:}_{3}\cdots\:{{\upgamma\:}}_{p}\right)$, is a column vector of unknown * p* regression parameters that are assumed to be the same for all individuals in the study, which measure the influence of the covariates on the survival experience, and $\:exp\left({{\upgamma\:}}^{\mathrm{T}}{X}_{i}\right)$ characterizes how the hazard function changes as a function of subject covariates, failure times [28].

We compared exponential, Weibull, log-logistic, and log-normal Accelerated Failure Time (AFT) models [29–31]. The Likelihood ratio test (Hosmer and Lemeshow, 2000), and Akaike information criteria (AIC) Akaike (1987) model selection methods were used. To check the proportional hazards assumption of the Cox proportional hazard model of the survival model, the Schoenfeld residuals and the GLOBAL test were displayed.

## Results

A total of 853 cholera patients admitted to Cholera Treatment Centers (CTCs) in conflict-affected agropastoral zones of Ethiopia since January 2023 were included. The median age, pulse rate, systolic and diastolic blood pressure of patients were 25 years (IQR: 7–36), 90 (IQR: 80–102), 128 (IQR: 110–144), and 70 (IQR: 60–80), respectively, with 51.9% being male and 48.1% female. About 83.1% of patients were from rural areas. Approximately 39% of patients presented with severe dehydration, 31.9% with moderate, and 29.1% with mild dehydration. Vomiting was reported in 60.5% of cases. 89.3% had no exposure, and nearly all reported no recent travel history. Regarding environmental conditions, 70.7% of patients used unprotected water sources, and only 8.6% had access to functional latrines. Household infection control was implemented in 23.1% of cases. The overall recovery rate was 98.8%, and 1.2% of patients died during treatment.

### Kaplan–Meier estimates and log-rank tests

The Kaplan-Meier method showed that there were significant differences in recovery in all subgroups. The median time to recovery was 2 days in patients with a low or normal pulse rate and 3 days in patients with a high pulse rate (*p* = 0.03). Time to recovery was also significantly different in different age groups. The median time to recovery was 2 days in patients less than 40 years of age and 3 days in patients aged 41 years and above (*p* = 0.005). Time to recovery was also significantly different in patients with mild/moderate and severe degrees of dehydration. The median time to recovery was 2 days in patients with mild/moderate and 3 days in patients with severe degrees of dehydration (*p* < 2 × 10–16). The median time to recovery was 2 days in vaccinated patients and 3 days in unvaccinated patients (*p* = 3 × 10 − 7). The median time to recovery was also significantly higher in patients who had a history of exposure (3 days) compared to patients without a history of exposure (2 days) (*p* = 0.01). Table 1 shows the number of events, median time to recovery, and the log-rank test for all subgroups. The above results show that both clinical and exposure-related factors are associated with time to recovery in patients.

**Table 1:** Median time to recovery, number of events, and log-rank test results for patient subgroups, showing significant differences in time to recovery by clinical and exposure-related factors

Variables	Subgroup	Number of events	Median recovery times (days)	Log-rank tests
Pulse rate	Low Normal High	135 608 110	2 2 3	0.03
Age of the patient	< 1 year 1–20 21–40 41–60 60 or older	105 276 298 141 30	2 2 2 3 3	0.005
Dehydration	Mild Moderate Sever	248 272 333	2 2 3	< 2e-16
Vaccination	Yes No	113 740	2 3	3e-07
Exposure history	Yes No	91 762	3 2	0.01

**Fig. 1:**
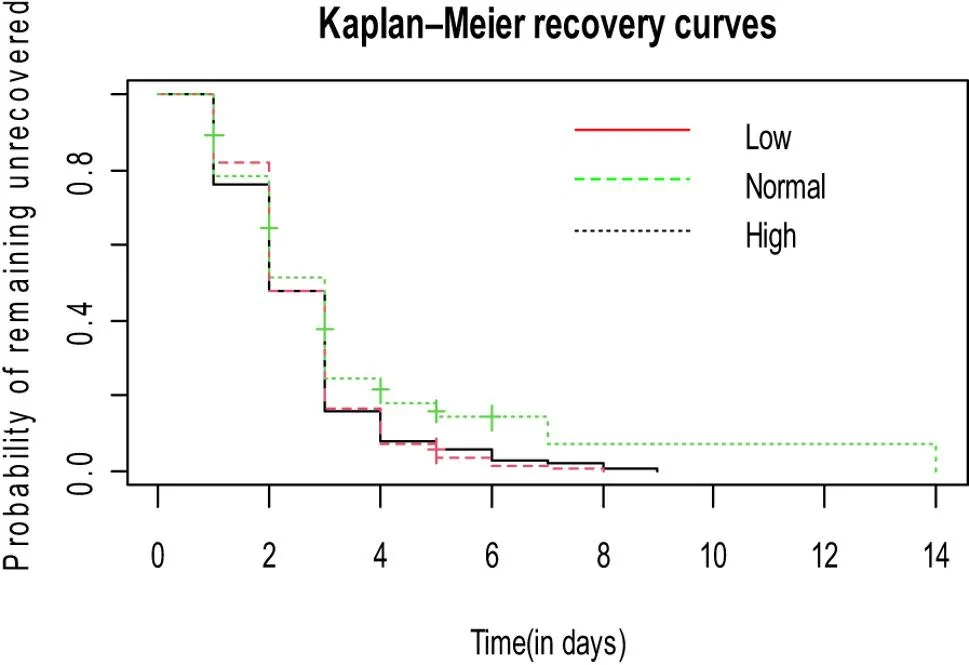
Kepler-Meier time-to-recovery curves for pulse rate

Kaplan-Meier plots were also stratified according to pulse rates, which were categorized into low, normal, and high, as depicted in Fig. 1. Patients who exhibited abnormal pulse rates, especially those who exhibited high pulse rates, showed a higher decrease in time to recovery rates compared to those who exhibited normal pulse rates. This indicates that tachycardia, which could be a symptom of acute dehydration and systemic illness, is associated with a longer recovery period.

**Fig. 2:**
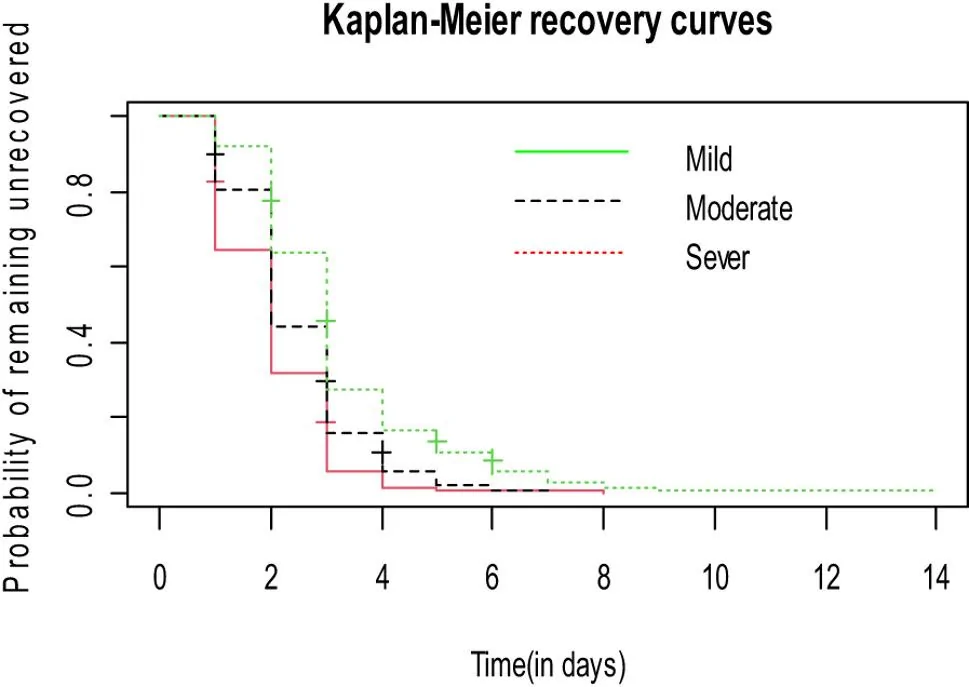
Kepler-Meier time-to-recovery cures for dehydration

Figure 2 shows the recovery curve for patients who exhibited mild, moderate, and severe dehydration. Patients who exhibited severe dehydration showed a higher likelihood of experiencing delayed recovery over a longer period compared to those who exhibited mild dehydration. The curve for mildly dehydrated patients showed a steeper fall, indicating a faster rate of recovery.

**Fig. 3:**
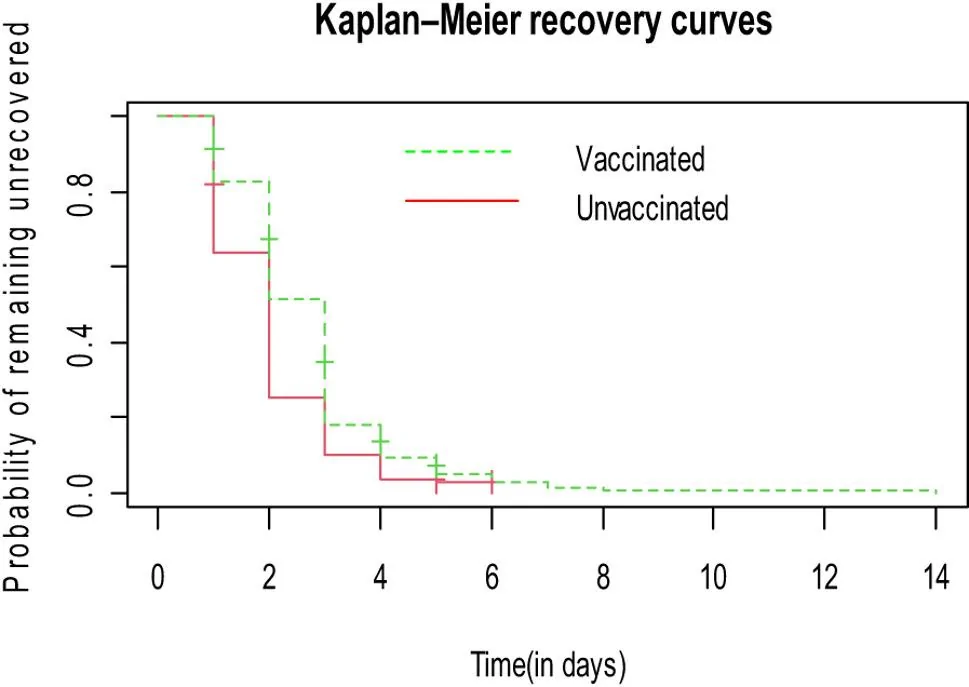
Kaplan-Meier time-to-recovery curve for vaccination

Figure 3 illustrates a comparison between the time-to-recovery curve for vaccinated and unvaccinated patients. The results showed that vaccinated patients recovered faster than unvaccinated patients, as depicted by a steeper curve for vaccinated patients, indicating a shorter recovery period. This could be an indication of the preventive role played by oral cholera immunization, which reduces the severity of illness and hence clinical outcomes.

**Fig. 4:**
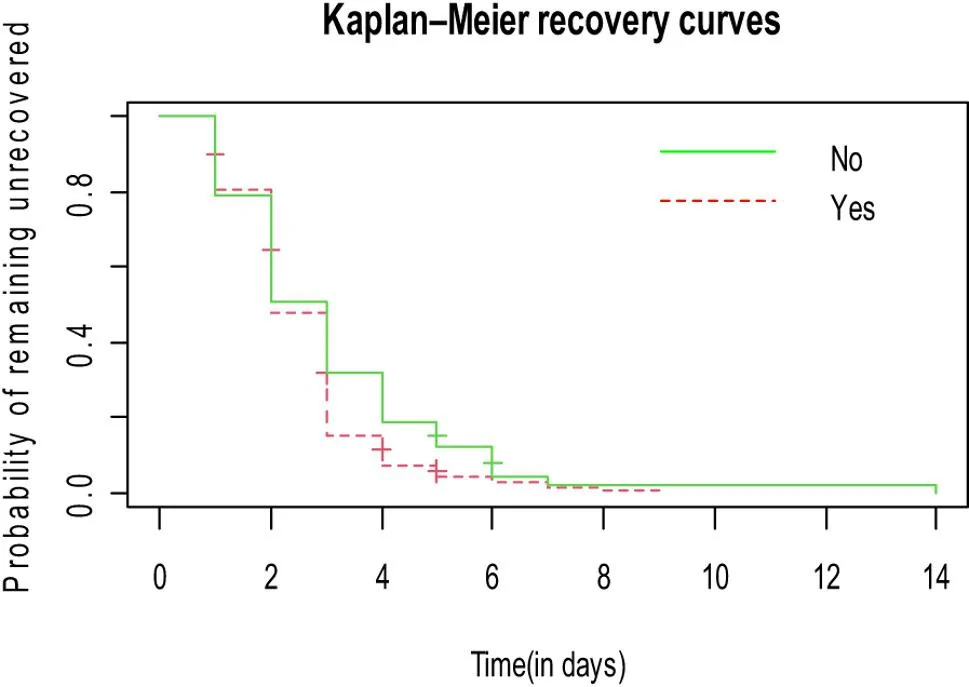
kaplan-Meier time-to-recovery curves for exposure history

Figure 4 demonstrates a comparison between therecovery periods for patients who exhibited and thosewho did not exhibit an exposure history. Patients whoexhibited an exposure history showed a slower rate ofrecovery compared to those who did not exhibit an exposurehistory. This could be attributed to repeated infections,delayed care-seeking behaviors, and sensitivity due transmission.

### Cox proportional hazards regression

Patients with a low pulse rate (HR = 0.69, *p* = 0.006) experienced a delayed recovery compared to those with a normal pulse rate in Table 2. A low pulse rate could indicate hypovolemia or severe dehydration, or possibly electrolyte imbalances, which may delay recovery. It might also reflect underlying bradycardia or poor perfusion, requiring more supportive care. Patients presenting with bradycardia may need closer monitoring, fluid resuscitation, and correction of electrolyte imbalances to hasten recovery.

A high systolic blood pressure (HR = 1.30, *p* = 0.001) was associated with faster recovery in Table 2, indicating that patients with higher BP were more likely to recover sooner. Because cholera frequently causes hypotension due to fluid loss, higher blood pressure may indicate better hydration status and less severe disease, helping to facilitate faster recovery. Age group 41–60 years (HR = 0.69, *p* = 0.025) showed slower recovery compared to the age group less than 1 year. Middle-aged adults may face longer recovery times, possibly due to comorbidities or delayed health-seeking behavior. Tailored interventions may be needed for this group.

Both moderate (HR = 0.68, *p* < 0.0001) and severe (HR = 0.49, *p* < 0.0001) dehydration were strongly associated with longer recovery times, with severe dehydration having the strongest negative impact in Table 2. These findings emphasize that dehydration severity is a critical determinant of cholera recovery. Rapid and aggressive fluid replacement remains essential to reduce recovery time.

Consistent with the expected protective effect of vaccination, unvaccinated individuals (HR = 0.57, *p* < 0.0001) had longer recovery times than vaccinated patients in Table 2. Vaccination may reduce disease severity and facilitate quicker convalescence. Patients with known contact with cholera cases (HR = 0.63, *p* = 0.0003) had a slower recovery process, suggesting that these individuals may have been exposed to a higher infectious dose or developed more severe illness due to delayed symptom recognition or care-seeking. This finding highlights the importance of targeted education and early monitoring of contacts during cholera outbreaks to promote timely treatment and reduce the duration and severity of illness.

**Table 2:** Result of cox proportional hazards model

Variable	Hazard ratio (95% CI)	*p*-value
Low pulse rate	0.69 (0.53, 0.90)	0.005
High systolic blood pressure	1.29 (1.11, 1.51)	0.001
Age 41–60 years	0.69 (0.50, 0.95)	0.03
Moderate (some) dehydration	0.68 (0.56, 0.81)	3.62e-05
Severe dehydration	0.49 (0.40, 0.59)	1.06e-13
Unvaccinated	0.57 (0.44, 0.73)	8.24e-06
Contact with suspected case	0.63 (0.49, 0.81)	0.0003

The Schoenfeld residuals test confirmed that the proportional hazards assumption was met (global test *p* = 0.28). The final model had a good fit with AIC = 9533.15.

### Parametric analysis result

The maximum likelihood of the unknown parameters for the log-normal distribution AFT model was better with a lower log- likelihood ratio (LR) test and AIC score − 1247 and 2521.95, respectively. The results of the measures showed that the selected covariates model can predict future observations, given that it best fits the data at hand.

The Log-Normal AFT model was suitable because the recovery time is skewed and has a log-normal distribution. The fitted Log-Normal AFT model identified several statistically significant covariates associated with recovery time. The recovery time cholera cases with low pulse rate, age 41–60 years, moderate dehydration, severe dehydration, unvaccinated, and contact with suspected case (exposure history) were 13%, 19%, 24%, 49%, 32%, and 29% longer recovery times compared to cases with normal pulse rate, age group less than 1 year, no dehydration, vaccinated and no contact with suspected cases respectively, while cases with high systolic blood pressure was 12% shorter compared to normal systolic blood pressure. The scale parameter (0.47) suggests moderate dispersion of recovery times in Table 3.

**Table 3:** Results of log normal distribution AFT model

Variable	Log-time ratio	*p*-value
Intercept	1.38	9.8e-05
Low pulse rate	1.13	0.05
High systolic blood pressure	0.88	0.0003
Age 41–60 years	1.19	0.02
Moderate (some) dehydration	1.24	3.1e-07
Severe dehydration	1.49	< 2e-16
Unvaccinated	1.32	4.5e-07
Contact with suspected case	1.29	1.2e-05
Log(scale)	0.47	< 2e-16
Scale	1.59	

## Discussions

In this study, the Log-Normal Accelerated Failure Time model was the most appropriate for analyzing cholera recovery time, as the data were positively skewed and best fit a log-normal distribution. This differs from findings in a Nigerian study, where the log-normal model provided a poorer fit based on the Akaike Information Criterion (AIC) [24]. This discrepancy may be attributed to differences in sample characteristics, such as the timing of event occurrence or sample size, which can influence model fit and assumptions about the underlying hazard function.

Several factors were identified as significant predictors of recovery. Patients with low pulse rates had a longer recovery than those with normal rates. A low pulse rate may reflect underlying bradycardia, which could indicate compromised cardiovascular function or advanced disease severity, potentially reducing the body’s physiological capacity to respond to infection and fluid loss. In contrast, higher systolic blood pressure was associated with faster recovery. Elevated systolic pressure could reflect better baseline circulatory status, suggesting that patients were better able to maintain perfusion and respond to treatment, particularly rehydration therapy. Efficient circulation aids in the rapid distribution of fluids and electrolytes, facilitating recovery. Patients with a known history of contact with cholera cases experienced slower recovery. This may be due to a higher infectious dose acquired through close exposure, which can result in more severe disease and longer recovery. Additionally, such individuals may delay seeking treatment due to misattributing early symptoms or underestimating disease severity, thereby worsening outcomes.

Age was another important determinant. Patients aged 41–60 years recovered more slowly than those less than 1 year of age. While infants are typically perceived as more vulnerable, middle-aged adults may experience worse outcomes due to the presence of comorbid conditions such as hypertension, diabetes, or reduced immune responsiveness. These underlying health issues can delay physiological recovery even when medical treatment is available. Previous studies also highlight that older age is associated with increased hospital stay duration [21] and greater risk of death [3, 22]. Furthermore, the Weibull model in a separate study found that patients aged 41–60 years had shorter survival times than those aged 21–40 years [24], reinforcing the notion that middle age may be a critical period of vulnerability due to a combination of physiological and comorbid risks [32].

Oral cholera immunization is a key strategy in reducing the disease burden and achieving positive clinical outcomes. Alongside prompt fluid resuscitation and effective antibiotic treatment, a sufficient OCV stockpile is a key requirement in the reactive response to outbreaks and in immunizing at-risk populations in high-risk settings [6, 12]. There is evidence that household immunity is achievable, as indicated by an estimated 54% protective effectiveness in persons residing in OCV-immunized households [16], and studies have indicated that vaccination in improved WASH environments could confer greater and longer-term protection against cholera transmission [17, 18]. OCVs remain an effective preventive strategy in high-risk groups during epidemics [6]. This is in line with the results, as vaccination status was found to have a significant effect on the recovery time in the current study, where unvaccinated persons took longer in recovery, while vaccinated persons showed faster recovery. This could be attributed to the lesser severity of disease, as indicated by recent studies that have demonstrated increased survival and significant reductions in mortality due to cholera in vaccinated persons [24, 32, 33].

The degree of dehydration at presentation had a profound effect on recovery time. Both moderate and severe dehydration were associated with prolonged recovery, with severe dehydration having the most detrimental impact. This is expected, as dehydration is a direct consequence of the massive fluid loss caused by cholera. Severe dehydration can impair organ perfusion, lead to electrolyte imbalances, and cause complications such as acute kidney injury, all of which contribute to delayed recovery. Previous studies have similarly shown that severe dehydration is strongly associated with increased mortality risk [3, 22]. Patients with severe dehydration had shorter survival times compared to those with moderate dehydration, while mildly dehydrated patients had better survival outcomes [24, 32], and [33]. These findings emphasize the importance of early detection and aggressive fluid replacement therapy, which are crucial for improving recovery trajectories and reducing fatality rates.

The study has a few limitations, first being the potential violation of the proportional hazards assumption of the Cox model, as well as the assumption in the log-normal AFT model that the recovery time distribution has to be correctly specified—an issue that may have influenced the results despite the Kaplan-Meier analysis supporting these results. Using secondary data is an additional limitation, where there is a potential for measurement or recording errors. It should be noted that this analysis was completed for a specific problem area, forming a limitation in generalizing these results to a much wider sphere. Additionally, although not addressed in this study, comorbid conditions, time-to-care, and treatment adherence influence the outcome and overall performance of the model.

## Conclusion and recommendations

This study identified several key clinical and epidemiological factors associated with time to recovery from cholera, using both Cox proportional hazards and Log-Normal Accelerated Failure Time (AFT) models, supported by Kaplan-Meier recovery analysis. The models consistently demonstrated that severe dehydration, lack of vaccination, exposure history, and low pulse rate were significant predictors of delayed recovery, while high systolic blood pressure and vaccination were associated with faster recovery. Overall, these findings support the continued scale-up of vaccination, early clinical assessment (including hydration and vital signs), and public health interventions targeting exposed individuals to improve outcomes and reduce transmission.

## Data Availability

The datasets created and analyzed during the current investigation are made available upon reasonable request from the authors.

## Abbreviations

AFT: Accelerated failure time
AIC: Akaike’s information criterion
AWD: Acute water diarrhea
CTC: Cholera treatment centers
EPHI: Ethiopian public health institute
WASH: Water, sanitation, and hygiene
WHO: World health organization
